# TumorMet: A repository of tumor metabolic networks derived from context-specific Genome-Scale Metabolic Models

**DOI:** 10.1038/s41597-022-01702-x

**Published:** 2022-10-07

**Authors:** Ilaria Granata, Ichcha Manipur, Maurizio Giordano, Lucia Maddalena, Mario Rosario Guarracino

**Affiliations:** 1 National Research Council, Napoli, 80131 Italy; 2 University of Cassino and Southern Lazio, Cassino, 03043 Italy

**Keywords:** Biochemical reaction networks, Cancer

## Abstract

Studies about the metabolic alterations during tumorigenesis have increased our knowledge of the underlying mechanisms and consequences, which are important for diagnostic and therapeutic investigations. In this scenario and in the era of systems biology, metabolic networks have become a powerful tool to unravel the complexity of the cancer metabolic machinery and the heterogeneity of this disease. Here, we present TumorMet, a repository of tumor metabolic networks extracted from context-specific Genome-Scale Metabolic Models, as a benchmark for graph machine learning algorithms and network analyses. This repository has an extended scope for use in graph classification, clustering, community detection, and graph embedding studies. Along with the data, we developed and provided Met2Graph, an R package for creating three different types of metabolic graphs, depending on the desired nodes and edges: Metabolites-, Enzymes-, and Reactions-based graphs. This package allows the easy generation of datasets for downstream analysis.

## Abstract (summary)

**Table:** 

Measurement(s)	gene expression, metabolic relationships
Technology Type(s)	Genome Scale Metabolic Models; Computational network biology
Sample Characteristic - Organism	Homo sapiens

## Background & Summary

Cancer is a complex disease caused by a myriad of factors and characterized by an astonishing complexity of phenotypes and traits, which determine its wide heterogeneity, even among cells of a single tissue. Nonetheless, three key processes are shared by all cancer cells: proliferation, invasion, and metastasis. To fulfill these tasks, cancer cells need to reprogram their metabolic activities and cross-talk with their neighborhood^1,2^. This evidence gives the metabolism and its players a crucial role in cancer progression and, consequently, cancer research.

Among all the biological networks, the metabolic ones are particularly complex and highly interconnected. Still, they probably are the best characterized in terms of connections and those that better represent the genotype-phenotype associations^3^. According to this, the reconstruction of comprehensive networks through the integration of omics data into metabolic scaffolds is one of the tools preferred by the systems biology approach for investigating biological phenomena from a holistic point of view. The metabolic scaffolds are given by the Genome-Scale Metabolic Models (GSMs), built from multi-omics data integration, and carrying information concerning the genes/proteins with enzymatic activity, how they interact with bioactive compounds in the context of biochemical reactions, and how the metabolic interconnections change in different cells, tissues or specific conditions^4^. There is a great interest in exploiting these models to generate condition-specific graphs at the service of machine learning approaches. In the era of precision medicine, the main goal is to develop approaches and tools to face the well-known heterogeneity of physiological and pathological manifestations and provide focused solutions for specific conditions. Considering the disease cohort as a single group, including all the diagnosed patients, is a simplistic approach that does not contemplate any inter-samples heterogeneity due to genetic and environmental factors. While modern biology has accepted the intra-sample heterogeneity of single cells, it seems anachronistic to talk about disease- instead of patient-specific conditions. There are several studies that address the problem of heterogeneity by exploiting network-structured approaches^5–7^.

Metabolic networks are complex and can involve different metabolic players (i.e., metabolites, enzymes, reactions). Machine and deep learning frameworks allow extracting knowledge from the metabolic networks while dealing with their structural and relational complexity^4^. In the context of findability, accessibility, interoperability, and reusability (FAIR) principles^8^, providing benchmark datasets for comparing novel approaches and for the general advancement of a specific research domain is extremely important. Graph-structured data coupled with machine learning approaches are receiving growing interest^9–13^, and many benchmark datasets have been proposed in the context of biomedical graphs, especially derived from protein-protein interaction, chemical, imaging data^14–18^. To the best of our knowledge, metabolic networks based on context- and patient-specific metabolic models have not been provided so far. To fill this gap, here, we provide the TumorMet repository. TumorMet contains two main sets of networks depending on the models from which they derive: Tissue-derived networks generated starting from tissue-specific models and PDGSMMs-derived networks obtained using Patient-Derived Genome-Scale Metabolic Models (PDGSMMs). The interesting implications of using the metabolic networks are twofold, from both a computational and biological perspective. Their complexity in terms of nodes and connections, and the plasticity given by the multiple ways in which they can be generated, make them appealing for the proposal and validation of novel approaches in the context of computational graph-based research. In this work, we presented three alternatives, each focused on a specific set of metabolic players (i.e., metabolites, enzymes, and reactions). As demonstrated by^19^, reconstruction algorithms used to generate context-specific models present a bug which determines an underestimation of the molecular context. The model’s conversion into a network allows further contextualization by integrating context-specific data. Being aware that the networks we generated for TumorMet are just a portion of the possibilities, we provided the Met2Graph package to give the user the freedom to build the networks depending on specific needs. Met2Graph indeed implements a flexible process flow to build the metabolic graphs, can be easily integrated with user-customized functions, and provides several arguments to personalize the networks. Some of the networks in this dataset were used for assessing graphs classification, clustering, and embedding^20–23^, as well as for multimodal data analysis^24,25^, demonstrating their benefits. An exciting field of biological network usage is also represented by the application of node classification approaches aimed at predicting the essential genes, namely those genes crucial for an organism’s viability. Usually, the Protein-Protein Interaction (PPI) networks are exploited to this extent, based on the assumption that the topological centrality is correlated to a functional centrality. As hypothesized in^26^, one of the reasons why the PPI are the most used networks for this purpose could be their abundance compared to the other types, such as Metabolic networks, highlighting the importance of providing network datasets. Still, only physical interactions, additionally not contextualized, are insufficient to represent the genetic connections’ complexity^27^. Modern biology extensively uses networks to integrate and analyze data in a way in which organisms, tissues, or cells are considered systems. This perspective gives a crucial role to the connections among biological components, and the network-based analyses are exploited for making relevant biological inferences. The central role of metabolism in different aspects of pathophysiological mechanisms and their tune regulation make these networks particularly interesting for extracting knowledge and making predictions. For example, the analysis of hub nodes^28^ and the comparison of topological properties between different context-specific networks^29^ are valuable resources in diagnostic and prognostic markers investigation for precision medicine. Along with the data, we also provide an R package, Met2Graph, to create metabolic graphs starting from GSMs and gene expression data. The package can generate three types of graphs, depending on the desired nodes and edges: Metabolites-based graphs, where metabolites are nodes connected by reactant-product relationships and the edges can be weighted by expression values of the enzymes catalyzing the corresponding reactions; Enzymes-based graphs, where enzymes are nodes that are connected if they catalyze two reactions, each producing and consuming a specific metabolite; and Reactions-based graphs, with reactions as nodes connected if the metabolite produced by one is consumed by the other. TumorMet is deposited at figshare repository^30^ and the Met2Graph package used to generate it is available at the Met2Graph Github repository (https://github.com/cds-group/Met2Graph).

## Methods

**Fig. 1:**
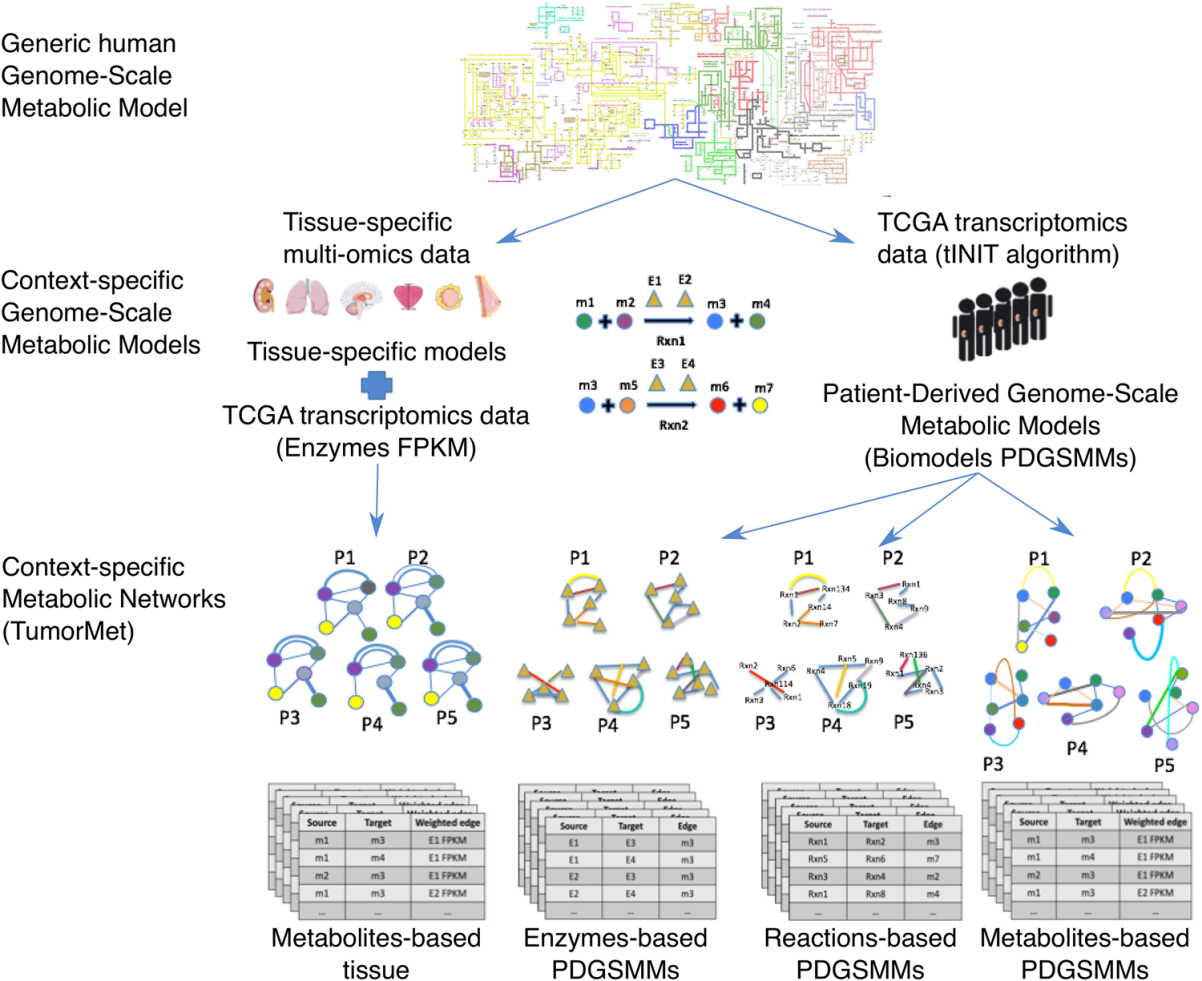
Overview of the Metabolic networks construction. The context-specific GSMs used in this study derive from the human generic GSM through the integration of tissue-specific multi-omics data (tissue-specific GSMs from Human Metabolic Atlas) or by integration of TCGA transcriptomics data (PDGSMMs from Biomodels). The context-specific GSMs carrying information about biochemical reactions are the input to create the context-specific metabolic networks of the TumorMet repository. Metabolites-based_tissue networks are generated by integrating TCGA gene/enzyme-expression data into the tissue-specific GSMs to weight the edges represented by enzymes connecting two metabolites. Networks of different patients have the same structure with different edge weights depending on patient expression profile. Enzymes-, Reactions and Metabolites-based_PDGSMMs networks are created from PDGSMMs and have enzymes/reactions as nodes connected by metabolites or metabolites as nodes connected by enzymes. Networks of different patients have different structures and no weights.

The metabolism involves several players, and focusing on one or another influences the type of analysis and the knowledge that can be extracted. The metabolites and the enzymes represent the main molecular components. A biochemical reaction is a transformation process that uses/consumes some metabolites (reactants) to produce new ones (products). The enzymes can facilitate these transformations as they are particular proteins having catalytic activity and the ability to speed up the rate of a reaction binding the substrate by a lock-key or induced-fit model. Not all the reactions are catalyzed by enzymes, as some of them can occur spontaneously. The enzymes are selective; this means that one binds specifically one or few substrates and, consequently, can catalyze one or more reactions, while the same reaction can be catalyzed by more enzymes acting as complex or as mutually exclusive catalyzers. This information is crucial in defining the rules to design a metabolic network since the connections between the metabolic players can be multiple and of different nature when involving the enzymes. In order to manage this issue, we defined some simplification strategies when enzymes represent edges and give rise to multiple connections (as in the case of Metabolites-based networks) and a different consideration of complex and mutually exclusive relationships when enzymes represent the nodes (as in the case of Enzymes-based networks). Further details are provided below in the network construction sections. The repository we provide contains different types of metabolic networks, depending on the nodes and the rules behind the connections: Metabolites-, Enzymes- and Reactions-based networks. A graphical overview of the metabolic networks construction is provided in Fig. 1.

### Metabolic models

Tissue-specific GSMs for 5 of the different origin sites of cancer (lung, kidney, brain, ovary, prostate)^31^ and breast cancer INIT model^32^ were downloaded from the Metabolic Atlas repository (http://www.metabolicatlas.org) in the compressed Systems Biology Markup Language (SBML) format^33^ to create the Metabolites-based graphs. PDGSMMs from the Biomodels repository (https://www.ebi.ac.uk/biomodels/pdgsmm/) have been downloaded to generate Metabolites-, Enzymes- and Reactions-based_PDGSMMs graphs for each patient. The Gene-Protein-Reaction (GPR) relationships were extracted from version 1.4.1 of the human generic GSM (https://github.com/SysBioChalmers/Human-GEM/tree/master/model).

### Gene expression data

Gene expression data from 6 different tumor primary sites were used to create context-specific Metabolites-based metabolic networks. FPKM (fragments per kilobase per million reads mapped) normalized and log-transformed read counts from RNA sequencing experiments of the breast (TCGA-BRCA), lung (TCGA-LUAD and TCGA-LUSC), kidney (TCGA-KIRC and TCGA-KIRP), brain (TCGA-GBM and TCGA-LGG), ovary (TCGA-OV), and prostate (TCGA-PRAD) cancers were obtained from the Genomic Data Commons (GDC) data portal (https://portal.gdc.cancer.gov). GDC includes several cancer projects, among which The Cancer Genome Atlas (TCGA), which we selected to download the data. Each of them represents a dataset of the repository. Clinical annotations of the samples were also extracted from the database and included in each dataset as sample-sheets.

### Metabolites-based_tissue networks construction

The metabolites are the nodes of the network, labeled by the corresponding ID, connected if they are involved in the same reaction, one as a reactant and one as a product. The connections have been created using the information from the relative context-specific metabolic model. Recurrent metabolites (e.g, ATP, CO2, H2O) have been removed to avoid redundant connections and unrealistic definition of paths^34^. The small molecules such as H2O, NH3, O2, CO2, phosphate, and cofactors are generally considered recurrent metabolites. The recurrent metabolites list we used is provided as external data of the package Met2Graph; the argument rmMets can be set to FALSE to avoid removal, or the list can obviously be personalized by the user. The GPR associations have been derived from the generic human GSM. Each edge is labeled by the Ensembl stable ID (in the form of ENS[species prefix][feature type prefix][a unique eleven-digit number]) of the enzyme/s catalyzing the reaction, when present, and weighted by the expression value/s of the corresponding gene/s obtained by the GDC Portal. Each resulting graph corresponds to a specific sample of the GDC tumor dataset considered. These rules create graphs where a couple of nodes can have multiple edges since multiple enzymes are involved in the same reaction and/or because the same nodes pair can be present in different reactions. Multiple edges have been simplified by averaging the expression values of enzymes acting in the same reaction and then summing up these averages corresponding to different reactions with the same nodes pair. Thus, all the graphs resulting from the same metabolic model have the same number of nodes and edges but different edge weights. The networks are then personalized for each patient by using the expression values and as a consequence, the gene context mentioned by^19^ is met. Based on the rules defining the edges, these networks are directed. The properties of these networks are summarized in Table 1.

**Table 1:** Properties of the Metabolites-based networks derived from tissue models.

	Kidney	Lung	Brain	Breast	Ovary	Prostate
# Graphs	928	1135	702	1217	379	551
# Vertices	4034	3990	3922	3394	3827	3939
# Edges	9210	9058	8914	6548	8533	8747
Edge density	0.000566	0.00056	0.00058	0.00057	0.00058	0.00056
Avg. network degree	4.56	4.54	4.54	3.86	4.46	4.44
Edge weights	√	√	√	√	√	√
Assortativity degree	−0.038	−0.035	−0.034	−0.049	−0.027	−0.03
Global transitivity	0.12	0.13	0.13	0.053	0.135	0.132
Avg. local transitivity	0.14	0.14	0.15	0.13	0.14	0.15
Minimum diameter	134.75	134.15	143.41	141.43	131.15	146.73
Maximum diameter	243.08	206.47	200.47	236.41	188.13	225.4

For each tissue dataset (along the columns), we report the number of graphs (first row) and the corresponding networks topological properties, such as the number of vertices and edges, edge density, average network degree, eventual presence of edge weights, assortativity degree, global transitivity, average local transitivity, minimum and maximum diameter (second through and eleventh rows). Observe that, for each tissue dataset, Metabolites-based networks share the same network structure, and thus topological properties, for all the samples since they derive from the same tissue metabolic model personalized with gene expression values.

### Metabolites-based_PDGSMMs networks construction

The logic behind the generation of Metabolites-based_PDGSMMs networks is the same as that of the networks derived from tissue models described in the previous paragraph, with the only difference that here each patient-specific network is derived from the corresponding PDGSMM downloaded from the BioModels repository. The edges are weighted using the patient’s gene expression data from the GDC repository. Therefore, each patient-specific network has a different structure and different edge weights. These graphs are directed and weighted. The properties of these networks are summarized in Table 2a.

**Table 2:** For each tissue dataset of the Metabolites- (a), Enzymes- (b), and Reactions-based_PDGSMMs (c) networks (along the columns), we report the number of graphs (first row) and the corresponding networks topological properties, such as the number of vertices and edges, edge density, average network degree, eventual presence of edge weights, assortativity degree, global transitivity, average local transitivity, minimum and maximum diameter (second through and eleventh rows).

	Kidney	Lung	Brain	Breast	Ovary	Prostate
**(a) Properties of the Metabolites-based networks from PDGSMMs**						
# Graphs	737	829	138	920	295	470
# Vertices	2679.05 ± 316.11	2619.5 ± 310.49	2634.49 ± 277.2	2576 ± 303.92	2576.93 ± 307.47	2676.14 ± 300.88
# Edges	6121.64 ± 839.57	6008.53 ± 908.15	6074.34 ± 783.77	5870.26 ± 841.16	5729.2 ± 837.64	5799.38 ± 769.08
Edge density	0.00086 ± 0.000009	0.0009 ± 0.0000009	0.0009 ± 0.0000009	0.0009 ± 0.0001	0.00087 ± 0.0001	0.0008 ± 0.000009
Avg. network degree	4.56 ± 0.23	4.57 ± 0.3	4.6 ± 0.28	4.54 ± 0.28	4.44 ± 0.29	4.33 ± 0.27
Edge weights	✓	✓	✓	✓	✓	✓
Assortativity degree	−0.01 ± 0.02	0.006 ± 0.029	−0.004 ± 0.033	0.012 ± 0.031	−0.008 ± 0.034	−0.018 ± 0.03
Global transitivity	0.16 ± 0.02	0.17 ± 0.026	0.16 ± 0.03	0.17 ± 0.029	0.14 ± 0.035	0.12 ± 0.035
Avg. local transitivity	0.12 ± 0.02	0.12 ± 0.02	0.12 ± 0.02	0.12 ± 0.02	0.11 ± 0.021	0.11 ± 0.02
Minimum diameter	134.8	134.01	140.76	118.44	120.84	145.97
Maximum diameter	302.7	241.72	217.06	255.8	211.24	282.75
**(b) Properties of the Enzymes-based networks from PDGSMMs**						
# Graphs	737	829	138	920	295	470
# Vertices	1941.256 ± 300.92	1859.76 ± 317.84	1911.35 ± 274.35	1846.58 ± 305.48	1859.98 ± 309.68	1934.25 ± 266.7
# Edges	63906.79 ± 18916.49	59341.79 ± 20947.88	63485.08 ± 17933.19	59530.08 ± 19744.67	59316.15 ± 202888.06	63922 ± 16898.25
Edge density	0.016 ± 0.002	0.016 ± 0.002	0.07 ± 0.002	0.016 ± 0.002	0.016 ± 0.002	0.016 ± 0.002
Avg. network degree	63.8 ± 14.16	61.23 ± 16.3	64.63 ± 14.07	62.15 ± 15.67	61.39 ± 15.86	64.49 ± 13.05
Edge weights	x	x	x	x	x	x
Assortativity degree	0.25 ± 0.04	0.25 ± 0.04	0.25 ± 0.04	0.26 ± 0.04	0.24 ± 0.046	0.25 ± 0.038
Global transitivity	0.18 ± 0.04	0.19 ± 0.046	0.18 ± 0.039	0.19 ± 0.046	0.19 ± 0.047	0.18 ± 0.035
Avg. local transitivity	0.29 ± 0.018	0.3 ± 0.02	0.03 ± 0.018	0.3 ± 0.02	0.298 ± 0.02	0.29 ± 0.015
Minimum diameter	14	13	13	14	13	14
Maximum diameter	34	36	28	33	35	30
**(c) Properties of the Reactions-based networks from PDGSMMs**						
# Graphs	737	829	138	920	295	470
# Vertices	3578.24 ± 595.037	3511.46 ± 637.32	3560.4 ± 543.41	3431.28 ± 591.12	3327.51 ± 582.49	3398 ± 527.44
# Edges	54823.89 ± 16130.9	60808.68 ± 19146.22	60137 ± 17749	59467 ± 18330	49776.08 ± 17158.5	46345.11 ± 14345.88
Edge density	0.0043 ± 0.0008	0.0048 ± 0.0007	0.004 ± 0.00085	0.0049 ± 0.00086	0.004 ± 0.00092	0.004 ± 0.0008
Avg. network degree	30.2 ± 6.13	33.74 ± 7.05	33.17 ± 7.1	33.9 ± 7.17	29.3 ± 6.96	26.91 ± 5.79
Edge weights	x	x	x	x	x	x
Assortativity degree	0.027 ± 0.016	0.052 ± 0.18	0.023 ± 0.15	0.048 ± 0.17	0.065 ± 0.2	0.06 ± 0.17
Global transitivity	0.028 ± 0.015	0.038 ± 0.017	0.037 ± 0.016	0.038 ± 0.017	0.028 ± 0.016	0.024 ± 0.013
Avg. local transitivity	0.038 ± 0.006	0.04 ± 0.006	0.043 ± 0.0059	0.04 ± 0.006	0.04 ± 0.006	0.04 ± 0.006
Minimum diameter	48	48	48	48	48	49
Maximum diameter	103	113	97	104	102	101

Observe that each network derived from PDGSMMs and corresponding to each patient sample has a different structure since the starting models are patient-specific (see Paragraphs on Metabolites-, Enzymes-, and Reactions-based PDGSMM networks). Therefore, values for network properties are reported as average ± standard deviation across all the networks of each dataset.

### Enzymes-based_PDGSMMs networks construction

These networks have enzymes as nodes connected if one catalyzes a reaction producing a metabolite consumed in a reaction catalyzed by the other. The recurring metabolites have also here been removed. According to the GPR, the enzymes involved in each reaction are associated by AND or OR logical relationship, indicating an enzymatic complex or an alternative activity, respectively. Based on this, enzymes related by AND have been considered as a single node, while OR relationships have been split into different nodes. To create patient-specific networks, PDGSMMs have been used as starting models for Metabolites-, Enzymes-, and Reactions-based_PDGSMMs datasets and downloaded from the BioModels repository. Each sample graph has then a different structure deriving from a different model. These graphs are directed and not weighted. The properties of these networks are summarized in Table 2b.

### Reactions-based_PDGSMMs networks construction

The rules behind these networks are similar to those of Enzymes-based networks, with the difference of having reactions as nodes, connected if one produces a metabolite consumed by the other. Recurring metabolites have been removed as well. To have sample-specific graphs also in this case we used the PDGSMMs from Biomodels. The resulting graphs are unweighted and directed, and each sample has a different structure determined by the different starting models. The properties of these networks are summarized in Table 2c.

### Simplified networks construction

Given the complexity and the size of these networks, we also provided a set of Metabolites-based sub-networks of a subset of kidney and lung samples, simplified according to the approach described in^21^. Briefly, central nodes have been selected by the Eigen centrality score, a measure describing the importance of a node in a graph that depends on that of its neighbors. The classification tests performed to demonstrate the reliability of these sub-networks compared to the whole networks gave comparable accuracy results (see Tables 3 and 4 in^21^). For each tissue, two sets of networks with a different number (#) of resulting nodes are provided. The properties of these networks, forming the Simpl-Kidney-# and Simpl-Lung-# datasets, are summarized in Tables 3 and 4.

**Table 3:** Properties of the Simplified Networks. See the caption of Table 1 for details.

	Simpl-Kidney-441	Simpl-Kidney-1034	Simpl-Lung-312	Simpl-Lung-1017
# Graphs	299	299	337	337
# Vertices	441	1034	312	1017
# Edges	1585	3226	1090	3102
Edge density	0.0163	0.006	0.022	0.006
Avg. network degree	7.18	6.24	6.98	6.1
Edge weights	✓	✓	✓	✓
Assortativity degree	−0.22	−0.13	−0.11	−0.12
Global transitivity	0.3	0.21	0.45	0.23
Avg. local transitivity	0.23	0.22	0.29	0.22
Minimum diameter	15.52	125.99	16.88	79.7
Maximum diameter	39.37	455.36	32.14	267.6

**Table 4:** Classes per dataset for usage validation of Metabolites-based networks through classification. Only primary tumors have been selected.

Kidney		Lung		Brain	
Cases	822	Cases	1025	Cases	666
Kidney Renal Papillary cell carcinoma (KIRP)	288	Adenocarcinoma (LUAD)	524	Glioblastoma multiforme (GBM)	155
Kidney Renal Clear cell carcinoma (KIRC)	534	Squamous cell carcinoma (LUSC)	501	Lower grade glioma (LGG)	511
**Breast**		**Ovary**		**Prostate**	
Cases	1085	Cases	290	Cases	497
		High-grade serous ovarian cancers subtypes^40^		Gleason score	
Basal-like	192	Differentiated	75	Pattern 3	199
HER2-enriched	82	Mesenchymal	75	Pattern 4	249
Luminal A	564	Proliferative	75	Pattern 5	49
Luminal B	207	Immunoreactive	65		
Normal-like	40				

### Classification

#### Metabolites-based_tissue datasets

In previous works, we have demonstrated the utility of the network datasets in classification and clustering tasks using subsets of some of the Metabolites-based graph datasets now included in the TumorMet repository^20,21,35–37^. Here, we extend to the entire repository the usage validation introduced in^20^, wherein we classify whole graphs sharing the same set of nodes. The basic idea is to 1) represent each graph of a dataset using probability distributions describing the topological properties of each node; 2) extract the distance matrix (Gram matrix), i.e., the symmetric square matrix containing the distances, taken pairwise, between the networks of the dataset; and 3) classify the networks based on the obtained distance vectors.

1. Based on the performance results achieved in^20,21,35–37^, here we selected the *Transition Matrix* of order one ${{\mathscr{T}}}^{r}$ for representing each graph ${{\mathscr{G}}}^{r}$, whose generic element ${{\mathscr{T}}}_{i,j}^{r}$ is the probability of a node *i* to be reached in one step by a random walker located in node *j*. Each row ${{\mathscr{T}}}_{i}^{r}$ of this matrix includes local information on the connectivity of node *i*.
2. For computing the distance between two networks ${{\mathscr{G}}}^{p}$ and ${{\mathscr{G}}}^{q}$, we selected the network distance:
  $${\mathscr{M}}({{\mathscr{G}}}^{p},{{\mathscr{G}}}^{q})=\frac{1}{l}{\sum }_{i=1}^{l}{d}_{JS}({{\mathscr{T}}}_{i}^{p},{{\mathscr{T}}}_{i}^{q}),$$
  obtained by averaging over all the *l* graph nodes the Jensen-Shannon distances *d*_*JS*_ of the probability distributions of their nodes^38^.
3. For classification, we considered the primary tumor classes described in Table 6. In particular, for Kidney, Lung, and Brain, the Primary-Tumor diagnoses indicated in the GDC sample metadata file, downloaded along with the gene expression files, have been used to label the samples and fulfill the classification task. For Breast, the 5 subtypes have been derived from the PAM50 classification^39^. As the Normal-like subtype has only 40 samples and is very similar to the Luminal A subtype, we performed the tests both including (Breast_5cl) and excluding (Breast_4cl) this class. For Prostate, as having only one class of diagnosis, the Gleason pattern score, an indicator of different grades of malignancy, has been used. Among the possible four classes (Pattern from 2 to 5), we excluded the Pattern 2 class (not shown in Table 6), as it is made of only one sample. Moreover, we considered two different classification problems: the Prostate1 case, that aims at discriminating the Pattern 3 samples (199) from the Pattern 4 ones (249); and the Prostate2 case, that consists in discriminating the Pattern 3 samples from the samples being assigned to Pattern either 4 or 5 (289). For Ovary, the subtype assignment of High-Grade Serous Ovarian Cancer (HGSOC) has been taken from^40^.

#### Metabolites-, Enzymes-, and Reactions-based_PDGSMMs datasets

The graph2vec framework^41^ is a neural method for learning graph-level embeddings in an unsupervised manner. It describes nodes through a recursive node relabeling algorithm assigning to each node a label uniquely representing its rooted subgraph (neighborhood). These labels form a vocabulary of words, and graphs are represented in the form of documents. Then, the Distributed Bag of Words doc2vec approach^42^ is used to learn the graph (document) embeddings. The performance has been evaluated by means of a stratified 10-fold Cross-Validation (CV) in which a SVM classifier, with a linear kernel, was applied to train and make predictions on 64-sized vectorizations of graphs (embeddings) produced by graph2vec with a recursive depth of 3 and a training duration of 200 epochs. The class labels used for the classification task are specified in Table 5.

**Table 5:** Classes of PDGSMMs used to accomplish the classification task of Kidney and Lung PDGSMMs derived networks.

	Kidney	Lung
Cases	737	829
Classes	KIRC 484	LUAD 429
	KIRP 253	LUSC 400

## Data Records

**Fig. 2:**
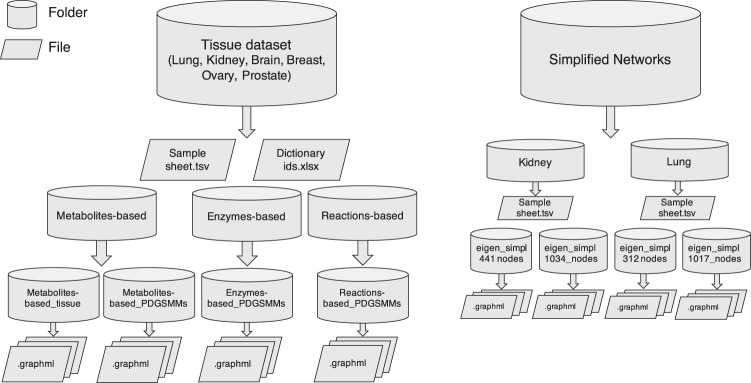
Scheme of the content of the TumorMet repository.

The network files and associated metadata composing the repository TumorMet are available at figshare repository^30^. The file TumorMet-repository.pdf summarizes the content of the repository. For easy access to the files, the repository is organized into seven datasets, each in a separate folder, representing the six tumor tissues and the simplified networks (i.e., Prostate, Lung, Kidney, Breast, Ovary, Brain, and Simplified networks). In each main tissue dataset folder, the sample-sheet file reporting the sample metadata as downloaded from GDC (i.e. Sample sheet.tsv) and an excel file reporting the correspondences between PDGSMM ids and TCGA ids (Dictionary_ids.xlsx) are provided. Each tissue dataset folder contains subfolders for the different types of networks, namely Metabolites-, Enzymes-, and Reactions-based, compressed in.zip format. The Metabolites-based folder is further subdivided into folders containing the Metabolites-based networks deriving from tissue models (Metabolites-based_tissue) and BioModels PDGSMMs (Metabolites-based_PDGSMMs). Enzymes- and Reactions-based networks are only derived from PDGSMMs. Simplified networks are provided for Kidney and Lung tissues. Each tissue folder contains the sample-sheet file reporting the sample metadata as downloaded from GDC (i.e., Sample sheet.tsv) and two subfolders for the networks files based on the number of nodes retained after the simplification process (for Kidney eigen_simplified_441_nodes and eigen_simplified_1034_nodes; for Lung eigen_simplified_312_nodes and eigen_simplified_1017_nodes). All the network files are provided in GraphML format. GraphML is a flexible and convenient XML format for storing network information. It supports unweighted, weighted, undirected, and directed networks and allows for the definition of node and edge attributes (http://graphml.graphdrawing.org/). A scheme of the repository content is illustrated in Fig. 2, while a summary of the networks features in terms of starting material and number of networks is provided in Table 6.

**Table 6:** Networks provided in the TumorMet repository.

	Type of network	Data used to build the networks	Number of networks
Kidney	Metabolites-based_tissue	• Tissue-Specific Model - Kidney • TCGA-KIRC & TCGA-KIRP GE	928: 607 TCGA-KIRC 321 TCGA-KIRP
	Metabolites-, Enzymes-, Reactions-based_PDGSMMs	• PDGSMMs from TCGA-KIRC & TCGA-KIRP • TCGA-KIRC & TCGA-KIRP GE (only for Metabolites-based)	737: 484 TCGA-KIRC 253 TCGA-KIRP
	Simplified	• Tissue-Specific Model - Kidney • TCGA-KIRC & TCGA-KIRP GE	299 for each simplification: 193 TCGA-KIRC 106 TCGA-KIRP
Lung	Metabolites-based_tissue	• Tissue-Specific Model - Lung • TCGA-LUAD & TCGA-LUSC GE	1135: 585 TCGA-KIRC 550 TCGA-KIRP
	Metabolites-, Enzymes-, Reactions-based_PDGSMMs	• PDGSMMs from TCGA-LUAD & TCGA-LUSC • TCGA-LUAD & TCGA-LUSC GE (only for Metabolites-based)	829: 429 TCGA-LUAD 400 TCGA-LUSC
	Simplified	• Tissue-Specific Model - Lung • TCGA-LUAD & TCGA-LUSC GE	337 for each simplification: 174 TCGA-LUAD 163-TCGA-LUSC
Brain	Metabolites-based_tissue	• Tissue-Specific Model - Brain • TCGA-GBM & TCGA-LGG GE	702: 173 TCGA-GBM 529 TCGA-LGG
	Metabolites-, Enzymes-, Reactions-based_PDGSMMs	• PDGSMMs from TCGA-GBM • TCGA-GBM GE (only for Metabolites-based)	138 TCGA-GBM
Breast	Metabolites-based_tissue	• INIT Cancer Model - Breast TCGA-BRCA GE	1217 TCGA-BRCA
	Metabolites-, Enzymes-, Reactions-based_PDGSMMs	• PDGSMMs from TCGA-BRCA • TCGA-BRCA GE (only for Metabolites-based)	920 TCGA-BRCA
Ovary	Metabolites-based_tissue	• Tissue-Specific Model - Ovary	379 TCGA-OV
		• TCGA-OV GE	
	Metabolites-, Enzymes-, Reactions-based_PDGSMMs	• PDGSMMs from TCGA-OV • TCGA-OV GE (only for Metabolites-based)	295 TCGA-OV
Prostate	Metabolites-based_tissue	• Tissue-Specific Model - Prostate • TCGA-PRAD GE	551 TCGA-PRAD
	Metabolites-, Enzymes-, Reactions-based_PDGSMMs	• PDGSMMs from TCGA-PRAD • TCGA-PRAD GE (only for Metabolites-based)	470 TCGA-PRAD

For each tumor tissue: the type of networks, the data used to generate the networks in terms of metabolic models and Gene Expression (GE) data from TCGA projects, and the number of networks, eventually subdivided by TCGA project ID. Observe that in the case of PDGSMMs derived networks, only for Metabolites-based_PDGSMM networks the GE data have been used to weight the edges.

## Technical Validation

Our validation process consisted of data-type and structural validation, as well as usage validation through downstream applications.

### Data-type and structural validation

The quality of the original data used to generate the networks is given by the reliability of the data sources repositories, i.e., GDC, Human Metabolic Atlas, and BioModels. Node IDs were verified to be of the same type. All edges were verified to be between nodes in the node list. All attribute data were verified to correspond to an existing node or edge. The structural integrity of the networks has been assessed by removing self-loops. Any duplicate edges were also removed. We further checked that nodes with no edges were not present in the networks.

### Usage validation

The tumor metabolic networks can be exploited in several downstream applications, ranging from pure network analysis to multi-level integration with other biological networks or data, to machine and deep learning approaches for unraveling the complex metabolic machinery and its role in precision medicine. In this section, we show the usage of TumorMet networks in classification of tumor samples, thus giving an idea of one of their potential applications. To furnish a baseline for comparing methods and approaches, we give several details of the two different workflows used for Metabolites-based networks derived from tissue models and Metabolites-, Enzymes-, Reactions-based networks derived from PDGSMMs.

#### Metabolites-based_tissue datasets

**Fig. 3:**
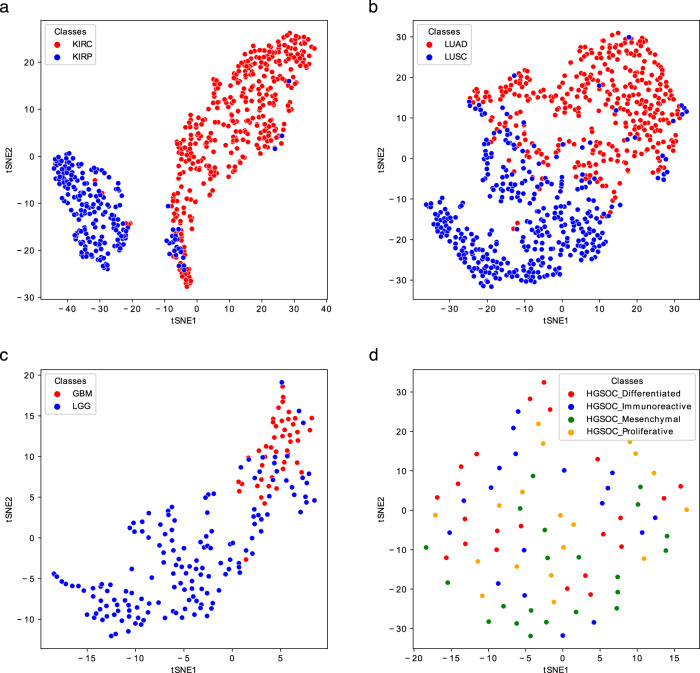
t-SNE representations of the Gram matrices of the test sets of the Kidney (**a**), Lung (**b**), Brain (**c**), and Ovary (**d**) Metabolites-based_tissue datasets. The TSNE function of the sklearn.manifold library has been used to generate the plots.

**Fig. 4:**
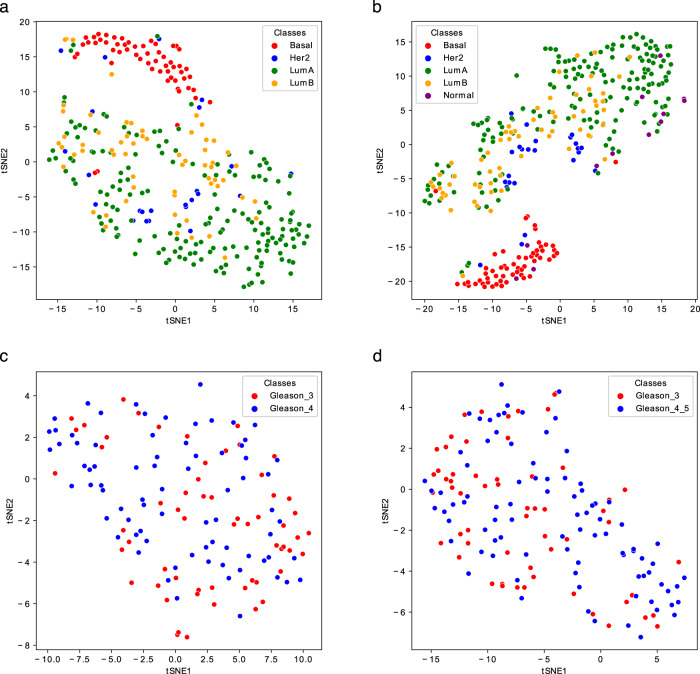
t-SNE representations of the Gram matrices of the test sets of the Breast_4cl (**a**), Breast_5cl (**b**), Prostate1 (**c**), and Prostate2 (**d**) Metabolites-based_tissue datasets. The TSNE function of the sklearn.manifold library has been used to generate the plots.
ii). For the statistical validation on the training sets, the data were min-max normalized and a Support Vector Machine (SVM) classifier with linear kernel was adopted using the libsvm implementation^43^ available in scikit-learn^44^. The one-vs.-rest strategy was used to classify the multi-class datasets. To account for unbalanced datasets, the “balanced” mode in sklearn was used to set the class weights; this parameter penalizes the wrong prediction of the classes having a number of instances lower than the others. The 10-fold CV on the training datasets was repeated 10 times, and the average of the CV scores are reported in Table 9 (top); these scores are also shown in the form of box plots in Fig. 5.

For the evaluation of classification performance, i) each of the Metabolites-based datasets was subdivided into a training and a test set; ii) a statistical validation was obtained on the training sets using a 10-fold CV, to ensure that the results were not biased to a specific training subset; iii) finally, the classification performance on the test datasets was evaluated using the models built on the training datasets.

i). In the case of Kidney, Lung, Breast, and Brain tissue datasets, the choice of the training sets was driven by our previous work^36^, where subsets of these datasets were already adopted for classification. Therefore, those subsets have been adopted here as training sets, while the newly added samples were assigned to the test sets. For the tissues not used previously (Ovary and Prostate), we obtained the training and test sets by using a 70:30 split ratio. The sample partitioning for each tissue is reported in Supplementary Table 1, while Figs. 3–4 provide the t-distributed Stochastic Neighbor Embedding (t-SNE) plots for the test sets.

**Fig. 5:**
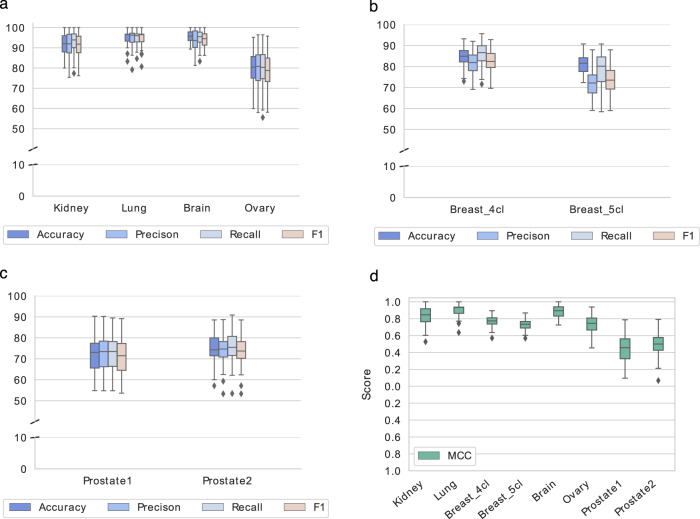
Classification scores on the Metabolites-based_tissue datasets. The box-plots show the classification scores obtained from the 10 iterations of the evaluation procedure on the training sets of the six Metabolites-based_tissue datasets. (**a**–**c**) report Accuracy, Precision, Recall, and F1 as percentages; (**d**) reports MCC values.
iii). The classification performance on the test sets was computed using the same SVM classifier learned on the training sets. The obtained results are reported in Table 9 (bottom). Kidney, Lung and Brain graphs are well classified, as shown by accuracy scores both in CV on training sets and using new samples as testing data (Table 9 and Figs. 3, 5). More challenging tasks are instead given by the classification of Breast, Ovary and Prostate samples.

Regarding Breast, the inclusion of the Normal-like subtype into the classification does not dramatically change the results; however, compared to the tissues mentioned above, the results are worse, having an accuracy of around 80%. Looking at the t-SNE plots (Fig. 4a,b), it is evident how the Basal is the best discriminated and most homogeneous subtype, while some samples of Luminal A, Luminal B, and Her2 are overlapped, especially the latter two. Normal-like samples, as expected, are difficult to separate from Luminal A ones. Ovary samples are completely overlapping (Fig. 3d) and lead to poor accuracy percentage (around 70%, as reported in Table 9). Finally, the CV scores reported in Table 9 (top) and plotted in Fig. 5c, as well as the test samples validation results reported in Table 9 (bottom), indicate that Prostate samples are generally poorly discriminated and the results are slightly better for the Prostate2 classification task (when the Gleason Pattern 5 is assimilated to Pattern 4). Prostate cancer is characterized by a high molecular heterogeneity^45^ which is evidently not caught considering only the Gleason score, as also highlighted by the t-SNE plots reported in Fig. 4c,d.

#### Metabolites-, Enzymes-, Reactions-based_PDGSMMs datasets

As detailed in the Section on Metabolic networks construction, these PDGSMMs derived graphs differ from the Metabolites-based graphs in that they do not share a common set of nodes across all patients. Therefore, we decided to accomplish the classification task on these datasets through a whole-graph embedding framework. Classification results based on these embeddings using the class labels specified in Table 5 for the Kidney and Lung PDGSMMs derived network datasets are reported in Table 8.

It is evident that the performance for these types of networks is not as good as the one obtained with Metabolites-based graphs, but it is worth pointing out that the two approaches to the classification task are completely different due to the different nature of the networks. Enzymes- and Reactions-based networks are indeed not weighted and have different structures being generated from different models. The complexity and density of these networks surely require a deeper investigation of the best suitable approach and parameters tuning to discriminate the differences among the samples, which is not the aim of this paper. As mentioned previously, one of the interesting aspects of the metabolic networks is their plasticity since different types of graphs can be generated depending on the desired nodes and connections. In future work, we will consider generating unique tri-partite graph for each patient to investigate the possibility to reduce classification performance differences. As for the networks extracted from tissue-specific models, the Metabolites-based_PDGSMMs networks are weighted by gene expression values. Comparing weighted vs. non-weighted networks in terms of classification performance, it is evident that the weights do not add any crucial information for discriminating the classes (Table 9). These networks derive from PDGSMMs reconstructed through the tINIT algorithm integrating TCGA gene expression data. Adding expression values to edges is therefore redundant and likely the models are already well contextualized. Instead, the weights have a different role in Metabolites-based_tissue networks, where are crucial for personalizing the networks in terms of patients. Furthermore, even if tested with different methods, the patients-specific Metabolites-based networks derived from tissue models seem to well contextualize the tissue models in terms of patients resulting as more representative of the tumor classes and with a higher discriminative power, as highlighted by classification performances (Table 7).

**Table 7:** Classification scores on Metabolites-based_tissue datasets.

# Classes	Kidney	Lung	Brain	Breast_4cl	Breast_5cl	Ovary	Prostate1	Prostate2
	2	2	2	4	5	4	2	2
**Cross-validation on training sets**								
# Samples per class	159/90	158/150	109/358	135/58/395/145	135/58/395/145/28	53/46/53/53	140/172	140/209
Accuracy avg %	92.80 ± 4.87	94.87 ± 3.68	95.83 ± 2.65	84.91 ± 4.15	81.02 ± 4.29	79.78 ± 7.79	71.83 ± 8.17	75.086.17
Precision avg %	91.97 ± 5.5	94.94 ± 3.85	93.63 ± 4.69	81.60 ± 4.99	72.30 ± 6.6	79.83 ± 8.57	72.23 ± 8.32	74.84 ± 6.11
Recall avg %	92.99 ± 5.1	94.95 ± 3.54	95.23 ± 3.57	85.93 ± 5.36	78.55 ± 7.3	79.93 ± 8.86	72.25 ± 8.22	75.99 ± 6.39
F1 avg %	92.12 ± 5.3	94.74 ± 3.83	94.15 ± 3.76	82.66 ± 4.76	73.36 ± 6.11	78.09 ± 8.83	71.14 ± 8.41	74.31 ± 6.35
MCC avg	0.85 ± 0.1	0.90 ± 0.07	0.89 ± 0.07	0.77 ± 0.06	0.73 ± 0.06	0.73 ± 0.1	0.44 ± 0.16	0.51 ± 0.12
**Test samples validation**								
# Samples per class	375/198	366/351	46/511	57/24/169/62	57/24/169/62/12	22/19/22/22	59/77	59/89
Accuracy %	97.03	93.72	91.00	85.26	83.64	70.59	73.53	73.00
Precision %	96.40	93.72	85.92	80.62	74.46	73.49	73.63	72.60
Recall %	97.13	93.72	93.36	83.99	81.83	70.33	74.05	73.53
F1%	96.75	93.72	88.56	82.02	77.48	71.05	73.44	72.57
MCC	0.94	0.87	0.79	0.77	0.76	0.61	0.48	0.46

Top: CV on training sets; Bottom: Validation on test sets.

**Table 8:** Classification scores on Enzymes- and Reactions-based_PDGSMMs Kidney and Lung datasets.

	Kidney		Lung	
	Enzymes-based_PDGSMMs	Reactions-based_PDGSMMs	Enzymes-based_PDGSMMs	Reactions-based_PDGSMMs
# Classes	2	2	2	2
# Samples per class	484/253	484/253	429/400	429/400
Accuracy avg %	78.97 ± 5.15	83.44 ± 4.32	78.17 ± 2.89	77.93 ± 2.62
Precision avg %	77.35 ± 6.00	81.99 ± 4.91	78.57 ± 2.69	78.36 ± 2.44
Recall avg %	75.72 ± 5.36	81.16 ± 5.00	78.00 ± 3.04	77.83 ± 2.75
F1 avg %	76.18 ± 5.42	81.39 ± 4.94	78.05 ± 3.04	77.76 ± 2.75
MCC avg	0.53 ± 0.11	0.63 ± 0.10	0.57 ± 0.06	0.56 ± 0.05

**Table 9:** Classification scores on weighted and unweighted Metabolites-based_PDGSMMs networks of Kidney samples.

	Metabolites-based_PDGSMMs Kidney	
# Classes	2	
# Samples per class	484/253	
	**weighted**	**unweighted**
Accuracy avg %	83.45 ± 4.58	85.48 ± 3.12
Precision avg %	82.28 ± 5.13	84.43 ± 3.71
Recall avg %	80.87 ± 4.82	82.99 ± 3.36
F1 avg %	81.32 ± 5.08	83.59 ± 3.47
MCC avg	0.63 ± 0.10	0.67 ± 0.07

## Usage Notes

The networks presented here have been generated using the Met2Graph R package we developed (see the paragraph on “Code availability”). The model in SBML format is imported and read by the Met2Graph package through the function readSBMLmod from the sybilSBML^46^ package. Several checkpoints are included in the function to validate the model object before importing it, such as check of upper and lower bounds, GPR mapping, reactions’ ids, and presence of list of reactants and products. The code snippets of Listings 1–4 show Met2Graph functions and arguments used to obtain the different networks:

**Listing 1** Metabolites-based_tissue networks.

**Listing 2** Metabolites-based_PDGSMMs networks.

**Listing 3** Enzymes-based_PDGSMMs networks.

**Listing 4** Reactions-based_PDGSMMs networks.

There are several open-source network libraries that can be used to analyze and visualize the networks provided in GraphML format. Examples of network analysis and visualization software include NetworkX, igraph, Cytoscape, yEd and Gephi.

## Supplementary information

Supplementary Table 1

## Data Availability

The R package Met2Graph developed and used to generate the TumorMet datasets is publicly available at the Met2Graph Github repository (https://github.com/cds-group/Met2Graph). The package has a detailed tutorial to generate the networks. Met2Graph implements a flexible process flow to build graphs starting from a GSM and can be easily integrated with user-customized functions. It allows the creation of the three different types of graphs described, based on the selection of nodes, edges, and attributes: Metabolites-, Enzymes- and Reactions-based graphs. It allows integrating gene expression data into Metabolites-based graphs. It provides several options and parameters to customize the resulting graphs. To name a few: to create multiple or simplified edges (simplification is possible using three different methods), to remove recurring metabolites, to consider the double direction in case of reversible reactions, to generate graphs as directed or not, and to plot the networks. All the details and the different arguments are described in the package manual and “help” section of the related functions. The code to compute the distribution based distance measures and to obtain the simplified networks is also available at the GraphDistances Github repository (https://github.com/cds-group/GraphDistances).
