# Publisher Correction: TumorMet: A repository of tumor metabolic networks derived from context-specific Genome-Scale Metabolic Models

**DOI:** 10.1038/s41597-022-01765-w

**Published:** 2022-10-21

**Authors:** Ilaria Granata, Ichcha Manipur, Maurizio Giordano, Lucia Maddalena, Mario Rosario Guarracino

**Affiliations:** 1 National Research Council, Napoli, 80131 Italy; 2 University of Cassino and Southern Lazio, Cassino, 03043 Italy

**Keywords:** Biochemical reaction networks, Cancer

Correction to: *Scientific Data* 10.1038/s41597-022-01702-x, published online 07 October 2022

In this article the funding from ‘European Union PON “Ricerca e Innovazione 2014-2020” FSC – Project PON03PE_00060_5 MEDIA ’ was omitted.

In the Acknowledgements section, the second sentence was also incorrect and should have read ‘It was carried out also within the activities of the authors as members of the INdAM Research group GNCS and the ICAR-CNR INdAM Research Unit’.

The original article has been corrected.

